# Dr. Black's Article

**Published:** 1875-06

**Authors:** 


Dr. Black's Article on "Gold," which appeared in the May number of JOURNAL, was taken from the *Transactions of N. Y. Odontological Society*, but had previously appeared in the "*Dental Cosmos*" as part of the proceedings of the meeting.

